# Effects of Sacrificing Tensor Tympani Muscle Tendon When Manubrium of Malleus Is Foreshortened in Type I Tympanoplasty

**DOI:** 10.1155/2015/531296

**Published:** 2015-11-30

**Authors:** Sohil Vadiya

**Affiliations:** Pramukhswami Medical College and Shree Krishna Hospital, Karamsad, Gujarat 388325, India

## Abstract

The current study aims at observing effects of sacrificing the tensor tympani tendon when manubrium of malleus is foreshortened or retracted on graft uptake, hearing improvement, and occurrence of complications if any during type I tympanoplasty surgery for central perforations. 42 patients were included in group A where the tensor tendon was sectioned and 42 patients were included in group B where the tensor tympani tendon was retained and kept intact. Graft uptake rates are very good in both groups but hearing improvement was found significantly better in group A than group B. No unusual or undesired complications were seen in any of the cases. Sectioning of tensor tympani tendon is safe and effective procedure in cases where manubrium is foreshortened.

## 1. Introduction

It is not unusual to find medially retracted or foreshortened handle of malleus (manubrium) during tympanoplasty. Apart from posing difficulties in placement of graft during underlay technique, it can affect orientation during surgery as the manubrium is one of the important landmarks in middle ear. Sectioning of the tensor tympani tendon near the neck of malleus would lateralize the manubrium to a significant extent and add to mobility of malleus as well. Arviso and Todd Jr. [1] have studied adult crania without clinical otitis and concluded that foreshortened malleus is an anatomic variant, not a sign of pathology. The current study aims at evaluating results of type I tympanoplasty for central perforations where manubrium was found foreshortened preoperatively or during surgery and the tensor tympani tendon (TT) was cut during surgery and comparing these results with those cases where manubrium was foreshortened and TT was kept intact.

## 2. Material and Methods

**Figure 1:**
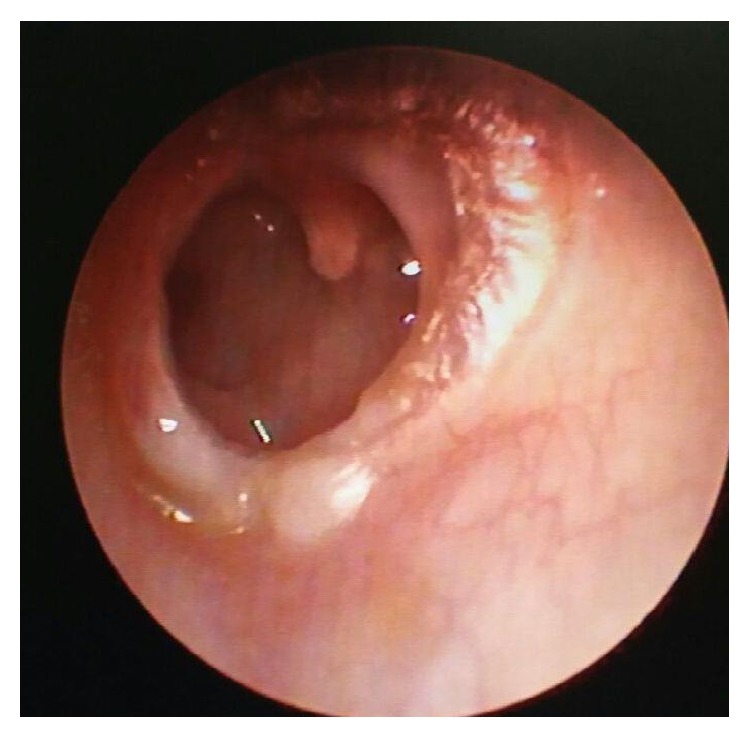
Foreshortened manubrium seen on otoendoscopy.

**Figure 2:**
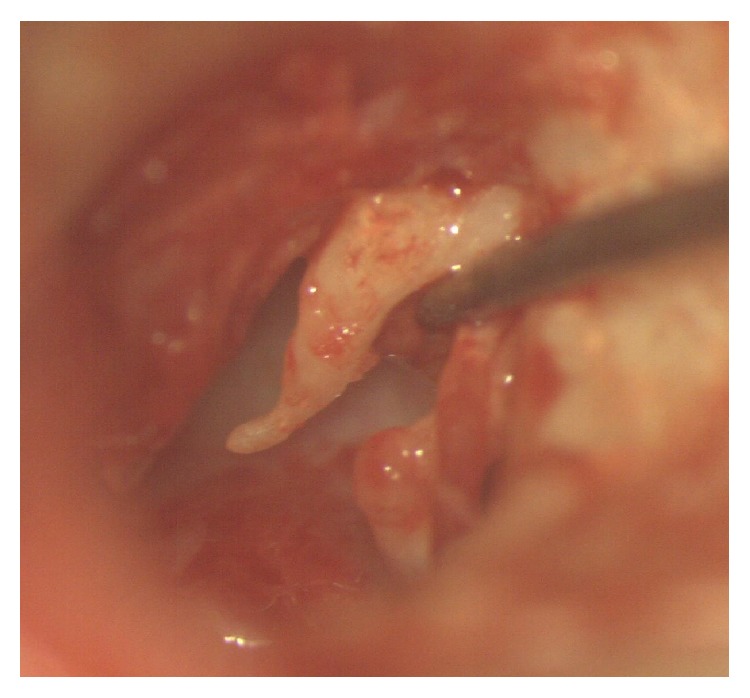
Tensor tympani tendon being cut during surgery.

A total of 84 cases were included in the study with inclusion criteria being a dry central perforation where the manubrium of malleus was found to be medially rotated and touching the medial wall of the middle ear. Cases with perforation size more than 4 mm (measured by placing graph paper on the perforation) are included. Cases where all the three ossicles were intact and mobile and where a type I tympanoplasty was performed were included. Cases with ossicular erosion or with cholesteatoma or with a marginal perforation were excluded. All subjects with mucosal chronic otitis media were clinically evaluated thoroughly including tuning fork tests and otoendoscopy done when ear is dry for more than 2 weeks. A pure tone audiogram was done for all the subjects. In some of them, a medially rotated malleus could be found during otoendoscopy (Figure 1) whereas, in many others, it was found during surgery. Odd numbered patients were included in group A where TT was cut during surgery (Figure 2). Even numbered patients were included in group B where TT was not cut. All cases were operated on under general anesthesia. Postauricular skin incision was used in all the cases in both groups. Vascular strip incision was used for canal wall skin and after middle ear contents were observed and after necessary disease removal, malleus was carefully made free of all attachments from remnant of tympanic membrane (TM). Ossicular mobility and intactness were also checked. If the subject met the inclusion criteria, decision of sectioning of TT was taken if the patient is in group A and TT was kept intact in patients of group B. Temporalis fascia was used as the graft material and kept lateral to the handle of malleus and medial to the annulus [2, 3]. Anterior tucking was done in all the cases in both groups. It was made sure during surgery that the annulus at the anterior canal wall is reposited back in the original position in the sulcus. Plenty of gelfoam was kept in middle ear, around ossicles especially medial to manubrium and also in the external ear canal in all cases. Cases were followed up regularly for the next 6 months minimum and audiometry results were recorded at 6 months postoperatively. Otoendoscopy picture at 6 months was taken into consideration. The same Amplaid A177 dual channel audiometer with standard calibration was used in all the cases to avoid errors. Parameters compared include graft uptake, medialisation suggested by graft touching the medial wall of middle ear, lateralisation suggested by blunting of anterior angle, air bone gap (ABG) at 6 months, and occurrence of squamous pearls.

## 3. Results

42 patients were included in group A where manubrium was found medialised and TT was cut near the neck of malleus and 42 patients belonged to group B where TT was not cut. All patients were between 20 and 40 years of age and there were 29 males and 13 females in group A and 27 males and 15 females in group B. There were 40 patients in group A where graft was successfully taken up and 2 cases where there was residual perforation at 8 weeks and they required revision surgery. In group B, graft uptake was complete in 39 patients and in one patient there was a tiny residual anterior perforation that healed with conservative management and two patients required revision surgery. So graft uptake rate is 95.24% for group A and 92.86% for group B. Medialisation was not seen in any patients in group A whereas 2 cases in group B developed graft medialisation where the neotympanic membrane was touching the promontory at 6 months postoperatively as evident on otoendoscopy. Blunting of anterior angle was not seen in any of the patients in both groups. The results of hearing thresholds are given in Table 1. Hearing thresholds at 500 Hz, 1000 Hz, and 2000 Hz were considered for hearing evaluation. The average preop ABG in group A was 35.60 db whereas in group B it was 36.76 db (*P* = 0.264). Average postop ABG in group A was 14.92 db and in group B was 19.88 db with statistically significant difference between the two groups (*P* < 0.000001) (Supplementary Material, available online at http://dx.doi.org/10.1155/2015/531296). Three cases in group A and 14 cases in group B had postoperative ABG more than 20 db. None of the patients in both groups had bone conduction threshold more than 15 db suggestive of sensorineural hearing loss. Average hearing gain in group A is 20.68 db whereas in group B it is 16.88 db. This shows clearly that hearing improvement in group A is significantly better than in group B.

Statistical analysis was performed with application of Student's *t*-test for the ABG values. No other complications were seen in any of the cases in both groups.

## 4. Discussion

Handle of malleus is longer than the long process of incus and this provides additional impedance matching function of middle ear and adds to improved conduction of sound through middle ear. When the handle is retracted severely, this should affect conduction of sound as well. Hol et al. [4] have used autologous interposition of incus to overcome severely retracted handle of malleus and stated that patients presenting with COM (chronic otitis media), a (central) perforation, a medially rotated malleus, and intact ossicular chain are a treatment challenge. Lateralizing the malleus handle may require disconnection of the ossicular chain and an autologous incus interposition to bring back the reconstructed tympanic membrane in its original position and improve the hearing. According to Todd [5], orientation of manubrium is inexplicably widely variable. Deng et al. [6] have concluded that the section of the tensor tympani muscle tendon in canal wall-down tympanoplasty with ossiculoplasty had no statistically significant influence on sound transmission and can be a safe maneuver in middle ear surgery.

It is well known, and we can see it in our cases; after cutting the tensor tympani tendon, the anterior tympanic membrane remnant becomes pleated, so we need to completely separate it from the manubrium. It will also make it easy to place the graft. The manubrium will support the graft from medial side, so the chances of medialisation should also be reduced. By cutting the tensor tympani tendon, the graft is more lateral thus increasing the middle ear volume. This will also help the ossicles to move more freely and it should improve hearing as adequate volume of middle ear is an important consideration for successful conduction of sound.

The current study aims to evaluate effect of sectioning the tensor tympani tendon in type I tympanoplasty surgery without mastoidectomy where the canal wall was preserved and the results are compared.  Table 2 shows comparison of graft uptake rates of different authors.

## 5. Conclusion

Graft uptake rates are adequate if tensor tympani is cut or preserved, whereas hearing improvements are better in patients where tensor tendon was cut and the difference is statistically significant. No other complications were observed in the current study in both groups. Sectioning of tensor tympani tendon is safe and effective procedure during tympanoplasty if manubrium is severely retracted and it brings good improvement in hearing also.

## Supplementary Material

The xl sheet shows post operative air bone gaps of cases in group A and group B. Student t test was used for statistical analysis and to calculate the *p* value.

## Figures and Tables

**Table 1:** Hearing evaluation in both groups (ABG: air bone gap).

Average ABG	Group A	Group B	Significance
Preop ABG (db)	35.60	36.76	*P* = 0.264
Postop ABG (db)	14.92	19.88	*P* < 0.00001
Hearing gain (db)	20.68	16.88	*P* < 0.00001

**Table 2:** Comparison of graft uptake rates of different authors.

Author	Graft material	Take-up (%)
Dabholkar et al. [7]	Temporalis fascia	84
Dornhoffer [8]	Perichondrium	85
Indorewala [9]	Fascia lata	95
Indorewala [9]	Temporalis fascia	66
Batni and Goyal [10]	Temporalis fascia	88
Present series	Temporalis fascia group A	95.24
Present series	Temporalis fascia group B	92.86
